# From hope to health: improving sexual and reproductive health knowledge among adolescent orphan girls through the ‘HopeBox’ intervention in Bangladesh

**DOI:** 10.7189/jogh.16.04312

**Published:** 2026-10-02

**Authors:** Fariya Rahman, Rokaiya Rokaiya, Samina Naznin, Mohammad Hridoy Patwary, Syed Moshfiqur Rahman, Lubna Hossain, Yeashir Arafath, Zara Munir, Abu Sayeed, Shanjida Momtaz, Saraban Tahura Ether, Abu Bakkar Siddique, Nusaibah Munir, Md Manjur Hossain, Md Shamsul Hoque, Maliha Mohsin, Quamrun Nahar, Shams El Arifeen, Ahmed Ehsanur Rahman, Anisuddin Ahmed

**Affiliations:** 1 Maternal and Child Health Division, International Centre for Diarrhoeal Disease Research, Bangladesh, Dhaka, Bangladesh; 2 Research and Learning, BBC Media Action, Dhaka, Bangladesh; 3 Health Systems and Population Studies Division, International Centre for Diarrhoeal Disease Research, Bangladesh, Dhaka, Bangladesh; 4 Department of Women's and Children's Health, Uppsala University, Uppsala, Sweden; 5 Department of Human Biology, University of Toronto, Toronto, Canada; 6 Department of Molecular Genetics, University of Toronto, Toronto, Canada; 7 Maternal and Child Health Services Unit, Directorate General of Family Planning, The Government of the People's Republic of Bangladesh, Dhaka, Bangladesh; 8 Maternal, Neonatal, Child, and Adolescent Health, Directorate General of Health Services, The Government of the People's Republic of Bangladesh, Dhaka, Bangladesh; 9 Department of Gender Studies, Central European University, Vienna, Austria; 10 James P Grant School of Public Health, BRAC University, Dhaka, Bangladesh

**Keywords:** sexual and reproductive health, HopeBox, adolescent, orphan, knowledge, Bangladesh

## Abstract

**Background:**

Orphaned adolescent girls face critical barriers to accessing accurate sexual and reproductive health (SRH) knowledge, which predisposes them to risky sexual behaviours and their consequences. We aimed to evaluate the effectiveness of the ‘HopeBox’ intervention in improving SRH knowledge among orphaned adolescent girls living in orphanages in Bangladesh.

**Methods:**

We adopted a pre- and post-test design to conduct the study from January 2022 to September 2025 among orphaned adolescent girls in three government-run orphanages in Dhaka, Faridpur, and Chuadanga, Bangladesh. We enrolled 173 eligible participants. We performed descriptive analyses to explore the distribution of sociodemographic variables and SRH indicators. We used McNemar’s tests to assess significant changes before and after the intervention. Generalised estimating equations with Firth penalisation were used to evaluate intervention effects after adjusting for relevant covariates and addressing data separation issues.

**Results:**

All five SRH knowledge indicators improved significantly: menstruation (34.73%), pregnancy avoidance (36.42%), contraceptive methods (37%), correct contraceptive use (32.92%), and sexually transmitted diseases (STDs) prevention (12.71%). After adjusting for covariates, participants in the post-intervention phase had substantially higher odds of demonstrating knowledge across all indicators, including menstruation (adjusted odds ratio (aOR) = 17.77; 95% confidence interval (CI) = 7.75–40.75), pregnancy avoidance (aOR = 21.86; 95% CI = 9.73–49.14), contraceptive methods (aOR = 41.73; 95% CI = 15.74–110.65), correct contraceptive use (aOR = 165.40; 95% CI = 28.42–962.79), and STDs prevention (aOR = 26.28; 95% CI = 5.65–122.23).

**Conclusions:**

The ‘HopeBox’ intervention improved SRH knowledge among orphaned adolescent girls, highlighting the need to scale up ‘HopeBox’ for orphan and vulnerable adolescents in Bangladesh to ensure equitable, appropriate, and accurate SRH knowledge.

Adolescents, defined by the World Health Organization as individuals aged 10–19 years [1], undergo puberty during this period, accompanied by significant physical, psychological, and emotional changes [2]. Irrespective of their gender, they face various challenges during their transition to adulthood, including issues related to sexual and reproductive health (SRH) [3]. Studies demonstrate that adolescents are a frequently underserved population in SRH, with this neglect influenced by multiple factors, including age-related characteristics and the sensitive nature of SRH [2]. These challenges are particularly pronounced among adolescents from vulnerable population groups, such as orphans [2].

Internationally, UNICEF classifies orphans into different categories, including single orphans (*i.e.* a child who has lost one parent), double orphans (*i.e.* a child who has lost both parents), and total orphans (*i.e.* a child who has lost mother or father) [4]. However, in Bangladesh, an orphan is defined as ‘a boy or girl aged <18 years who has lost his or her father or has been abandoned by his or her parents or guardians’ [5]. Currently, there are 4.8 million orphans residing in Bangladesh [6]. Orphans generally have less access to reproductive health services than their non-orphan peers [7]. Though there is a lack of data from Bangladesh, a study done in Zimbabwe in 2006 reports that the majority of vulnerable orphan girls do not have general knowledge regarding reproductive health and rights and are more likely to engage in sex at a younger age and riskier behaviour than non-orphan girls [7]. The average age at first sex for the orphan girls was 13.2 years, whereas for non-orphan girls it was 13.8 years. They also had a conservative view regarding the use of contraception, mostly about condoms. Many participants in the study believed that a condom should be used when an individual is having sex with multiple partners or in exchange for money. As a result, only 14% of them used a condom the first time they had sex.

Orphan adolescents are more vulnerable to sexual abuse, coercion, and exploitation [8]. A study in Ethiopia suggested that orphans are vulnerable to early, risky, and forced sex and STIs, including HIV/AIDS, due to a lack of knowledge, understanding of SRH issues, and agency [9]. According to the study, almost 50% of orphans had an early debut to sexual activity before reaching the age of 15. Moreover, 84% were victims of forced sex, and 75% had multiple sexual partners. In addition, orphan girls were found to be more engaged in transactional sex than the non-orphan girls, and the rate of practising unprotected sex was also high among the orphan girls [10].

Several studies reported that orphan girls are more likely to be subject to deprivation of basic SRH necessities [7,9–11]. Thus, it predisposes orphan girls to the risk of unwanted pregnancy and life-threatening STIs (*e.g.* HIV/AIDs) [9]. Though SRH challenges faced by orphans were evaluated in several countries, data remain scarce, and the SRH knowledge and practice status of orphan adolescent girls in Bangladesh is vastly understudied [11]. The National Strategy for Adolescent Health in Bangladesh envisioned providing quality and age-appropriate information and services to adolescents to improve the SRH status of adolescents [12]. Despite these positive approaches, the existing national programmes lack focus on the special needs of orphan girls to address their critical social status. Moreover, there is a dearth of information about vulnerable adolescents, such as orphan adolescents’ awareness, their needs, access, and supply of SRH resources and services. Furthermore, incorporating SRH policies into practice also remains challenging, particularly for marginalised groups, as orphan girls encounter discrimination and unjust treatment during their development [12,13].

Previous studies demonstrated that age-appropriate comprehensive sexuality education has a positive impact on SRH [14-16]. Therefore, there is a need for an intervention that can deliver accurate and age-appropriate SRH information. To address this gap, the International Centre for Diarrhoeal Disease Research, Bangladesh (icddr,b), in collaboration with government and non-government stakeholders, designed the intervention ‘HopeBox’ to overcome common barriers to SRH knowledge through engaging and age-appropriate educational tools. Using pre- and post-intervention assessments, we conducted a pilot study to examine changes in SRH knowledge across five key domains. We aimed to evaluate whether the ‘HopeBox’ intervention can significantly improve SRH knowledge among adolescent girls residing in orphanages in Bangladesh.

## METHODS

### Study design

We used a pre- and post-test design to assess the ‘HopeBox’ intervention's effect. We conducted the study across three sites following a structured timeline for pre-intervention, intervention, and post-intervention phases. The study sites were the girls’ orphanages ‘*Shishu Paribar* (*Balika*)’ situated in Dhaka, Faridpur, and Chuadanga. We purposively selected the sites to capture the geographical variation. The study timeline was from January 2022 to September 2025. The intervention was introduced in three fields between September 2024 and early November 2024.

### Study population

The study population comprised orphan adolescent girls aged 10–19 years. We included all participants who formally provided consent to participate in the study. For minors aged 10–17, we used an assent form to obtain consent from guardians. We excluded participants who could not read Bengali.

### Sample size

We calculated the sample size using McNemar’s discordant proportion formula [17]. Assuming 90% statistical power, a 95% confidence level, a proportion of participants gaining knowledge post-intervention of 0.15 [18], no participants losing knowledge post-intervention (since educational interventions on SRHR among adolescents have consistently demonstrated knowledge gains without evidence of knowledge reduction in the existing literature) [18,19], a 10% non-response rate, and a 10% loss to follow-up, the final sample size was 173. Among them, 74 girls were from Dhaka, 75 from Faridpur, and 24 from Chuadanga.

### Intervention

‘HopeBox’ is an integrated, composite SRH intervention, designed to be age-appropriate and contextually relevant for adolescent girls residing in government-run children’s homes, considering their social and cultural realities. The ‘HopeBox’ includes five booklets, two puzzles, three postcards, and one menstruation tracking calendar (Figure S1, Table S1 in the **Online Supplementary Document**). The research team delivered ‘HopeBox’ to participants in the selected orphanages during the implementation phase. The research team conducted three extensive training sessions to explain the purpose and components of the ‘HopeBox’ and guide participants on how to engage with and use its materials. The components of ‘HopeBox’ are illustrative and instructional, designed to promote peer-to-peer communication and disseminate SRH knowledge effectively among orphan adolescent girls. The development process of this intervention followed a co-design approach, engaging multiple stakeholders, including adolescent girls residing in government orphanages, their care providers, SRH experts, women and child health specialists, physicians, professors, representatives from international development organisations, policymakers, and scientists from icddr,b, ensuring the content is relevant, accurate, and culturally appropriate.

### Study variables

To better understand the study population, we collected information on several sociodemographic and personal characteristics through a semi-structured questionnaire. These included participants’ age, education level, communication with relatives, and access to media. Following the guidance from the United States Agency for International Development framework [20], we constructed five composite indicators to evaluate participants’ SRH knowledge pre- and post-intervention. The indicators were:

1 Knowledge and conception about menstruation: participants were considered to have correct knowledge and conception about menstruation (scored ‘1’) if they could accurately answer the definition of menstruation, at what age girls generally start menstruating, the duration of menstruation, and at what age girls stop menstruating.
2 Knowledge about ways to avoid pregnancy: participants were asked if they were aware of modern contraceptive methods; those who responded accurately were considered to know about pregnancy avoidance.
3 Knowledge about methods of contraception: participants were asked if they knew about at least two contraceptive methods out of 14 modern contraceptive methods available in Bangladesh; those who responded were considered to know methods of contraception.
4 Knowledge about the correct use of contraceptives: regarding the knowledge about the correct use of contraceptives, two contraceptive methods – condom and oral contraceptive pill – were considered. If participants could correctly answer either how to properly use a condom or how many times an oral contraceptive pill can be ingested daily, they were considered to have correct knowledge.
5 Knowledge about ways to avoid sexually transmitted diseases (STDs): participants who answered correctly which contraceptive method can prevent STDs were considered to have correct knowledge.

### Statistical analysis

We used descriptive analysis to describe participants’ background characteristics, including age, educational level, communication with relatives, and access to media across study sites. We calculated percentage changes in knowledge before and after the intervention, and used the McNemar test to assess significant differences across all SRH indicators. To explore how these changes varied by participant characteristics, we performed McNemar tests or the exact McNemar test (for sparse data) within each variable category. To evaluate the intervention's effect on all SRH indicators, we fitted generalised estimating equations (GEE) with Firth-type penalisation and an exchangeable working correlation, adjusting for relevant covariates using a logit link. This penalisation was necessary to address complete separation in the data, which led to convergence issues and infinite estimates in standard GEE models [21].

We used *R*, version 4.4.1 (R Core Team, Vienna, Austria) for all analyses. The ‘geefirth’ package was used to fit GEE with Firth-type penalisation [22].

## RESULTS

We assessed 394 girls for eligibility; 256 met the criteria, and 175 received the ‘HopeBox’ intervention (**Figure 1**). During the study period, two participants from Chuadanga married and left the orphanage; therefore, we excluded them from the analysis because we could not collect endline data. As a result, 173 girls were finally analysed using a modified intention-to-treat approach [23].

**Figure 1:**
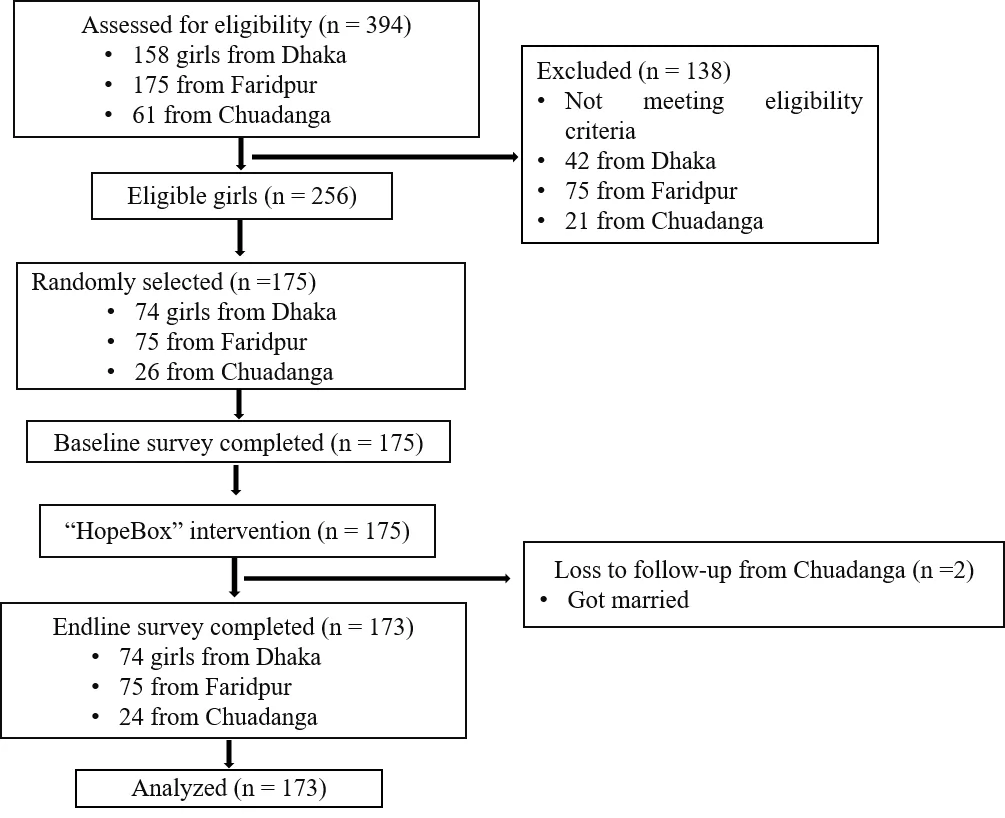
Participant flow diagram.

Among the 173 girls, 74 (42.77%) were from Dhaka, 75 (43.35%) from Faridpur, and 24 (13.87%) from Chuadanga (**Table 1**). Of the total participants, 68.2% were aged 10–14 years, while 31.8% were aged 15–19 years. Regarding education, most participants (46.51%) were at the secondary level. Overall, 85.5% of participants reported communicating with their relatives. Across study sites, 61.30% of participants from Faridpur, 95.80% from Chuadanga, and 31.10% from Dhaka had access to media every day.

**Table 1:** Overview of participant characteristics across study sites*

	Dhaka (n = 74)	Faridpur (n = 75)	Chuadanga (n = 24)	Total (n = 173)
**Age group in years**				
10–14	50 (67.60)	49 (65.30)	19 (79.20)	118 (68.20)
15–19	24 (32.40)	26 (34.70)	5 (20.80)	55 (31.80)
**Education**				
Primary	30 (40.50)	33 (44.0)	11 (45.80)	74 (43.02)
Secondary	38 (51.40)	32 (42.70)	10 (41.70)	80 (46.51)
Higher secondary	6 (8.11)	10 (13.30)	2 (8.33)	18 (10.47)
**Communication with relatives**				
Yes	63 (85.10)	64 (85.30)	21 (87.50)	148 (85.50)
**Access to media**				
Everyday	23 (31.10)	46 (61.30)	23 (95.80)	92 (53.20)
Occasionally	47 (63.50)	25 (33.30)	0 (0.0)	72 (41.60)
Not at all	4 (5.41)	4 (5.33)	1 (4.17)	9 (5.20)

*Values are presented as n (%).

After the intervention, knowledge and conception about menstruation increased by 34.73%, pregnancy avoidance by 36.42%, contraceptive methods by 37%, correct use of contraceptives by 32.92%, and STD prevention by 12.71% (**Figure 2**).

**Figure 2:**
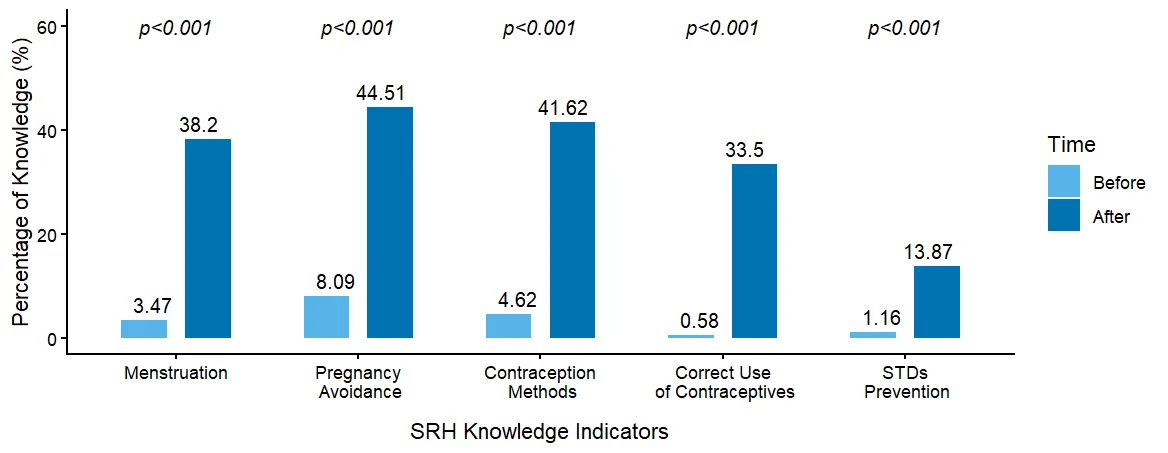
Percentage of SRH knowledge among orphaned adolescent girls before and after the intervention. SRH – sexual and reproductive health, STD – sexually transmitted disease.

Improvement percentages were higher across all five components for those aged 15–19 years than for those aged 10–14 years (**Table 2**). Specifically, for those aged 10–14 years, knowledge and conception about menstruation increased by 33.01%, pregnancy avoidance by 25.46%, contraception methods by 23.75%, correct use of contraceptives by 16.9%, and STDs prevention by 5.8%, while the for those aged 15–19 years, knowledge and conception about menstruation increased by 38.18%, pregnancy avoidance by 60%, contraception methods by 65.5%, correct use of contraceptives by 67.28%, and STDs prevention by 29.06%. The improvements also varied across study sites. For example, participants in Dhaka showed the greatest improvement in knowledge about pregnancy avoidance (43.3%), while those in Faridpur (40%) and Chuadanga (58.3%) showed the greatest improvements in knowledge and conception about menstruation. Regarding education, participants with secondary education showed greater percentage improvements than those with primary education. For example, those with primary education had an 18.32% improvement in knowledge about menstruation and a 1.64% improvement in the correct use of contraceptives, while those with secondary education showed a 46.2% improvement in knowledge about menstruation and a 43.9% improvement in the correct use of contraceptives. Improvements also varied by whether participants communicated with their relatives: those who did showed the largest rise in knowledge about menstruation (36.36%), while those who did not showed the largest rise in knowledge about contraceptive methods (47.9%). Lastly, regarding media access, participants with occasional media access exhibited higher percentages of improvement among all five components compared to those with regular media access. Among those with occasional media access, the improvement was the highest in knowledge about contraception methods (54.06%), while those with regular media access showed the highest improvement in knowledge about pregnancy avoidance (37.53%).

**Table 2:** SRH knowledge before and after the intervention by participant characteristics*

	Knowledge and conception about menstruation			Knowledge about pregnancy avoidance			Knowledge about contraception methods			Knowledge about correct use of contraceptives			Knowledge about STDs prevention		
	**Pre**	**Post**	***P*-value**	**Pre**	**Post**	***P*-value**	**Pre**	**Post**	***P*-value**	**Pre**	**Post**	***P*-value**	**Pre**	**Post**	***P*-value**
**Age group in years**															
10–14	2 (1.69)	41 (34.70)	<0.001	3 (2.54)	33 (28.0)	<0.001	1 (0.85)	29 (24.60)	<0.001	0 (0.0)	20 (16.90)	NA†	0 (0.0)	6 (5.08)	NA†
15–19	4 (7.27)	25 (45.45)	<0.001	11 (20.0)	44 (80.0)	<0.001	7 (12.70)	43 (78.20)	<0.001	1 (1.82)	38 (69.10)	<0.001	2 (3.64)	18 (32.70)	<0.001
**Study site**															
Dhaka	3 (4.05)	19 (25.70)	<0.001	8 (10.80)	40 (54.10)	<0.001	5 (6.76)	36 (48.60)	<0.001	1 (1.35)	30 (40.50)	<0.001	2 (2.70)	15 (20.30)	<0.01
Faridpur	3 (4.0)	33 (44.0)	<0.001	5 (6.67)	27 (36)	<0.001	2 (2.67)	26 (34.70)	<0.001	0 (0.0)	23 (30.70)	NA†	0 (0.0)	8 (10.70)	NA†
Chuadanga	0 (0.0)	14 (58.30)	NA†	1 (4.17)	10 (41.70)	<0.001	1 (4.17)	10 (41.70)	<0.01	0 (0.0)	5 (20.80)	NA†	0 (0.0)	1 (4.17)	NA†
**Education level**															
Primary	1 (1.35)	12 (19.67)	<0.001	0 (0.0)	6 (9.84)	NA†	0 (0.0)	4 (6.56)	NA†	0 (0.0)	1 (1.64)	<0.001	0 (0.0)	0 (0.0)	NA†
Secondary	4 (5.0)	42 (51.20)	<0.001	5 (6.25)	47 (57.30)	<0.001	1 (1.25)	45 (54.90)	<0.001	0 (0.0)	36 (43.90)	<0.001	0 (0.0)	13 (15.90)	NA†
Higher secondary	1 (5.56)	12 (41.40)	0.01	9 (50.0)	24 (82.80)	0.288	7 (38.90)	23 (79.30)	0.11	1 (5.56)	21 (72.40)	0.01	2 (11.10)	11 (37.90)	0.28
**Communication with relatives**															
No	0 (0.0)	7 (25.93)	NA†	3 (12.0)	15 (55.60)	<0.001	1 (4.0)	14 (51.90)	0.04	0 (0.0)	10 (37.0)	NA†	1 (4.0)	1 (3.70)	NA†
Yes	6 (4.05)	59 (40.41)	<0.001	11 (7.43)	62 (42.50)	0.07	7 (4.73)	58 (39.70)	<0.001	1 (0.67)	48 (32.90)	<0.001	1 (0.67)	23 (15.80)	<0.001
**Access to media**															
Not at all	0 (0.0)	2 (33.33)	NA†	2 (22.20)	2 (33.30)	NA†	2 (22.22)	2 (33.30)	NA†	0 (0.0)	2 (33.30)	NA†	1 (11.10)	1 (16.70)	NA†
Occasionally	4 (5.56)	14 (34.15)	0.28	10 (13.9)	25 (61.0)	<0.001	5 (6.94)	25 (61.0)	<0.01	1 (1.39)	22 (53.70)	<0.01	1 (1.39)	12 (29.30)	0.04
Everyday	2 (2.17)	50 (39.68)	<0.001	2 (2.17)	50 (39.70)	<0.001	1 (1.09)	45 (35.70)	<0.001	0 (0.0)	34 (27.0)	NA†	0 (0.0)	11 (8.73)	NA†

SRH – sexual and reproductive health, STD – sexually transmitted disease

*Values are presented as n (%).

†*P*-values of McNemar’s test or exact McNemar’s test could not be computed due to the absence of discordant pairs between pre- and post-intervention observations.

After adjusting for covariates (*i.e.* age, education level, study sites, communication with relatives, and access to media), we found that participants had higher odds of having knowledge about menstruation (adjusted odds ratio (aOR) = 17.77; 95% confidence interval (CI) = 7.75–40.75), pregnancy avoidance (aOR = 21.86; 95% CI = 9.73–49.14), contraception methods (aOR = 41.73; 95% CI = 15.74–110.65), correct use of contraceptives (aOR = 165.40; 95% CI = 28.42–962.79), and STDs prevention (aOR = 26.28; 95% CI = 5.65–122.23) in post-intervention phase compared to the pre-intervention phase (**Table 3**).

**Table 3:** Comparison of SRH knowledge among participants before and after the intervention

	aOR (95% CI)				
	**Knowledge and conception about menstruation**	**Knowledge about pregnancy avoidance**	**Knowledge about contraception methods**	**Knowledge about correct use of contraceptives**	**Knowledge about STDs prevention**
**Pre**	ref	ref	ref	ref	ref
**Post**	17.77 (7.75–40.75)	21.86 (9.73–49.14)	41.73 (15.74–110.65)	165.40 (28.42–962.79)	26.28 (5.65–122.23)

aOR – adjusted odds ratio, CI – confidence interval, SRH – sexual and reproductive health, STD – sexually transmitted disease, ref – reference

## DISCUSSION

We piloted whether the intervention ‘HopeBox’ improves SRH knowledge among orphaned adolescent girls in Bangladesh. We found that ‘HopeBox’ remarkably improved knowledge across the five specific SRH domains: menstruation, pregnancy avoidance, contraception methods, correct contraceptive use, and STDs prevention.

Among the five specific SRH indicators, the most substantial improvement was perceived in knowledge related to the pregnancy avoidance, contraception methods and their correct use. This reflects the generally low level of understanding among adolescents about how to protect themselves from unintended pregnancies and sexually transmitted infections. According to a study in Bangladesh, more than 60% of unmarried girls had knowledge about family planning methods [24], while another study reported that 40% were aware of them [25]. However, we found that only 8.06% knew how to avoid pregnancy, 4.62% had knowledge about contraceptive methods, and less than 1% knew how to correctly use contraceptives before our intervention. This shows a knowledge gap among orphan girls compared with their non-orphan peers. This gap may be attributed to the lack of parental guidance and family support, stigma, shame, and cultural taboos surrounding discussions of sexuality, which prevent them from accessing accurate information about SRH [26]. Although adolescents may be aware of contraceptive methods, their actual use remains low [27]. The low performance of contraceptive use among the adolescents in Bangladesh may be attributed to insufficient knowledge about sexuality and reproduction [27] and this limited knowledge about contraceptives may lead to unplanned pregnancies [28]. Therefore, the literature recommends continued efforts to raise awareness and promote proper contraceptive use among adolescents [29,30].

Another area the intervention improved was knowledge and conception about menstruation. Previous studies demonstrated the poor understanding of menstruation among adolescents in Bangladesh, with approximately half of adolescents lacking accurate knowledge or holding misconceptions [28,29]. In our study, only 3.47% of participants had knowledge of menstruation and its conception before the intervention, similar to findings from a study in Egypt, where only 8.5% of orphans had good knowledge of reproductive health [31]. This low level of knowledge may be because, in Bangladesh, mothers or other female relatives generally convey information about menstruation [30]. For orphaned girls, the absence of such familial guidance likely contributes to the markedly lower knowledge levels we observed [31].

We found that ‘HopeBox’ improved knowledge about the correct use of contraceptives the most, while the improvement in menstruation knowledge was comparatively lower. This is expected because all girls experience menstruation in their lives, so many of them might have some basic knowledge even before the intervention. As a result, the change in this area was smaller than in the other indicators. However, the aORs and their CIs of each indicator, modelled separately, showed a consistent pattern of improvement across all indicators. This shows that, although the level of improvement varied, ‘HopeBox’ consistently increased knowledge across all SRH indicators. This pattern of improvement suggests that ‘HopeBox’ was designed to be context-specific and age-appropriate, with accessible and relevant content that enables young people to confidently engage with a broad range of SRH topics.

We found that older orphanage adolescents (15–19 age group) had higher knowledge than the younger group (10–14 age group) before the intervention. The result aligns with previous studies showing that older adolescents generally possess greater SRH knowledge [32]. Since older teens have more independence and are better able to seek information and services, they are more likely to access SRH services, whereas younger adolescents often rely on guidance received from female relatives or guardians and have limited access to such resources [33]. The intervention increased knowledge across all five SRH indicators in both age groups, but the percentage increase was consistently higher in the 15–19 age group. Therefore, we can conclude that the ‘HopeBox’ intervention can serve as a reliable source of information for older adolescents and provide guidance to younger adolescents.

The percentage improvement in knowledge also varied across education levels. Girls with secondary or higher secondary education showed high improvement in all SRH knowledge indicators, consistent with earlier research findings [34]. This may be because more educated adolescents are generally more open to acquiring new knowledge and more likely to learn about available SRH services and their benefits [35,36]. However, as orphans, they often lack adequate resources to access accurate SRH information. The ‘HopeBox’ intervention provided this opportunity, enabling them to apply critical thinking and thereby achieve higher SRH knowledge. Additionally, we found ‘HopeBox’ to increase knowledge in all five components among those who did and those who did not communicate with their relatives. However, improvements in knowledge regarding pregnancy avoidance, contraceptive methods, and correct contraceptive use were greater among girls who did not communicate with their relatives. In Bangladesh, mothers often serve as the primary source of SRH information, especially for older adolescent girls [37]. Therefore, participants without parental guidance relied more on ‘HopeBox’ as a primary source of information.

Regarding media access, participants with occasional access showed greater improvement in SRH knowledge. This suggests that although they had some media exposure, they previously received limited information about SRH topics from those sources. The ‘HopeBox’ intervention likely filled this gap, indicating that combining ‘HopeBox’ with media access can lead to a greater enhancement of SRH knowledge.

Recent reviews of the literature and reports from organisations working with adolescents and youth in Bangladesh have concluded that adolescents lack access to accurate information about reproduction, fertility, SRH, and fertility regulation [38–40]. Moreover, their reproductive health needs are largely overlooked by current programmes. Adolescents’ knowledge and attitudes toward SRH services depend on their social and economic conditions and the availability of these services [32]. These findings underscore the urgent need for formal, age-appropriate, and culturally sensitive programmes like the ‘HopeBox’ intervention, which specifically address the emotional, informational, and developmental needs of vulnerable adolescents in institutional care.

This study has several strengths. First, this is the first study that explored the SRH issues of orphans in Bangladesh and focused on orphaned adolescent girls residing in institutional care, an underserved and often overlooked population. Second, we used a rigorous analytical approach that considered correlations within individuals, making the findings more reliable. Third, the assessment enabled a more comprehensive evaluation of the intervention’s impact by incorporating multiple dimensions of SRH knowledge. Finally, the pre-post design facilitated a direct comparison of participants’ knowledge before and after the intervention.

This study also has several limitations. First, we collected the sample from a limited number of institutions within specific geographic areas. This resulted in a relatively small sample size and restricted the generalisability of the findings. Second, we focused on girls living in orphanages; future studies should also focus on boys and adolescents outside institutional care. Third, the absence of a control group prevents clear separation of the intervention effect from potential time-related influences. Additionally, we conducted the follow-up assessment three months after the intervention. As a result, the findings may reflect short-term improvements in knowledge, and the long-term gains remain unclear. Again, as a pilot study, we did not have sufficient data to disentangle the distinct contributions of each component in each phase; therefore, developing a formal theory of change was beyond the scope of this study. Future research should incorporate longer follow-up periods to evaluate sustained knowledge and theory of change. Moreover, several subgroups had small sample sizes; as a result, observed differences in knowledge improvement across subgroups may reflect chance variation rather than actual effects. Furthermore, the distribution of outcome variables was skewed, which may have contributed to unstable model estimates and reduced the precision of the reported associations. Lastly, due to the inherently sensitive nature of SRH topics, some participants may have chosen not to respond or may have provided socially desirable rather than accurate answers, which was also observed in a previous study [41].

## CONCLUSIONS

Our findings suggest that exposure to the ‘HopeBox’ intervention is associated with significant improvements in SRH knowledge. Because this was a pilot study, larger, more rigorous studies with longer follow-up periods, behavioural change outcomes, and cost-effectiveness analyses are needed. If future evaluations continue to show promising results, ‘HopeBox’ may be considered for integration into national adolescent health strategies, including the Sarkari Shishu Paribar Management Policy 2002 (amended in 2023).

## Additional material


Online Supplementary Document

## Data Availability

**Data availability:** The data are available from the corresponding authors upon reasonable request.
