# Supplementary material for: From hope to health: improving sexual and reproductive health knowledge among adolescent orphan girls through the ‘HopeBox’ intervention in Bangladesh
Source: J Glob Health. 2026 Oct 2;16:04312. doi: 10.7189/jogh.16.04312 (PMC13631770; doi:10.7189/jogh.16.04312)
Supplement: Supplementary file 1 [file jogh-16-04312-s001.pdf]

Supplement to: Rahman F, Rokaiya R, Naznin S, Patwary MH, Rahman SM, Hossain L, Arafath Y, Munir Z, Sayeed A, Momtaz S, Ether ST, Siddique AB, Munir N, Hossain MM, Hoque MS, Mohsin M, Nahar Q, Arifeen SE, Rahman AE, Ahmed A. From hope to health: Improving sexual and reproductive health knowledge among adolescent orphan girls through the 'HopeBox' intervention in Bangladesh. J Glob Health. 2026;16:04312.

“HopeBox”

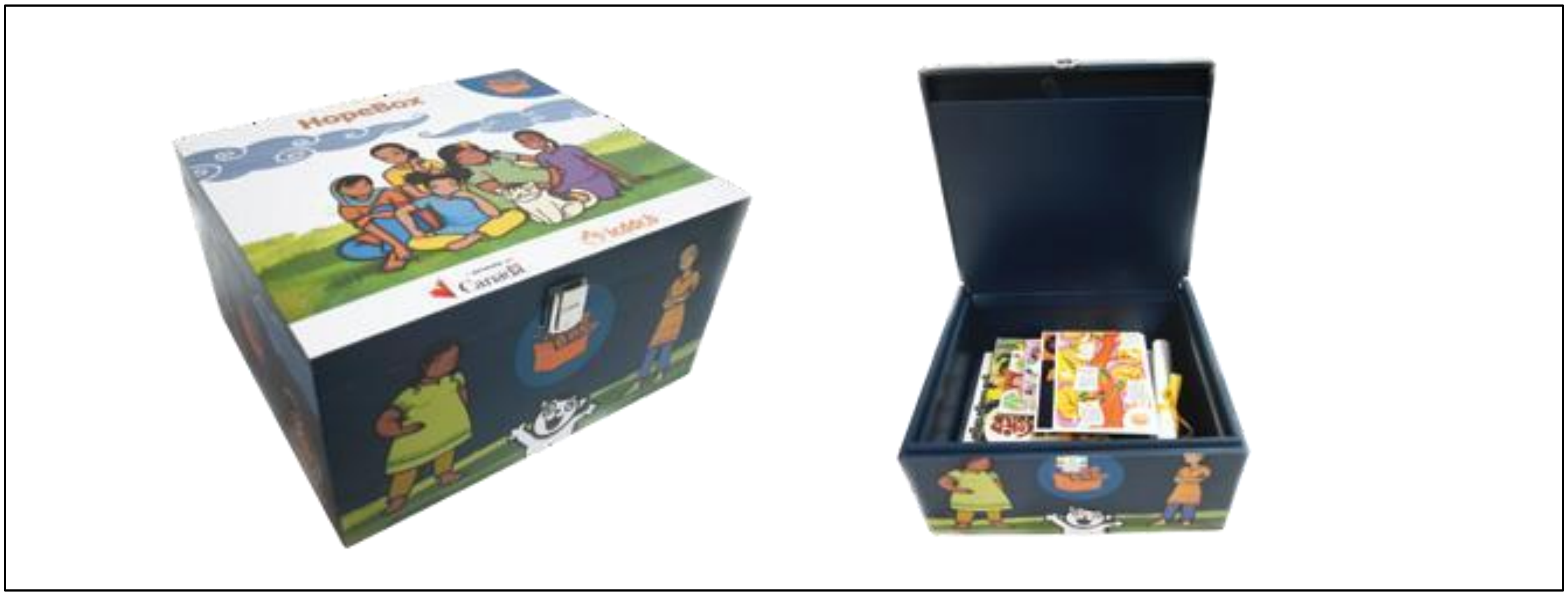

Booklets

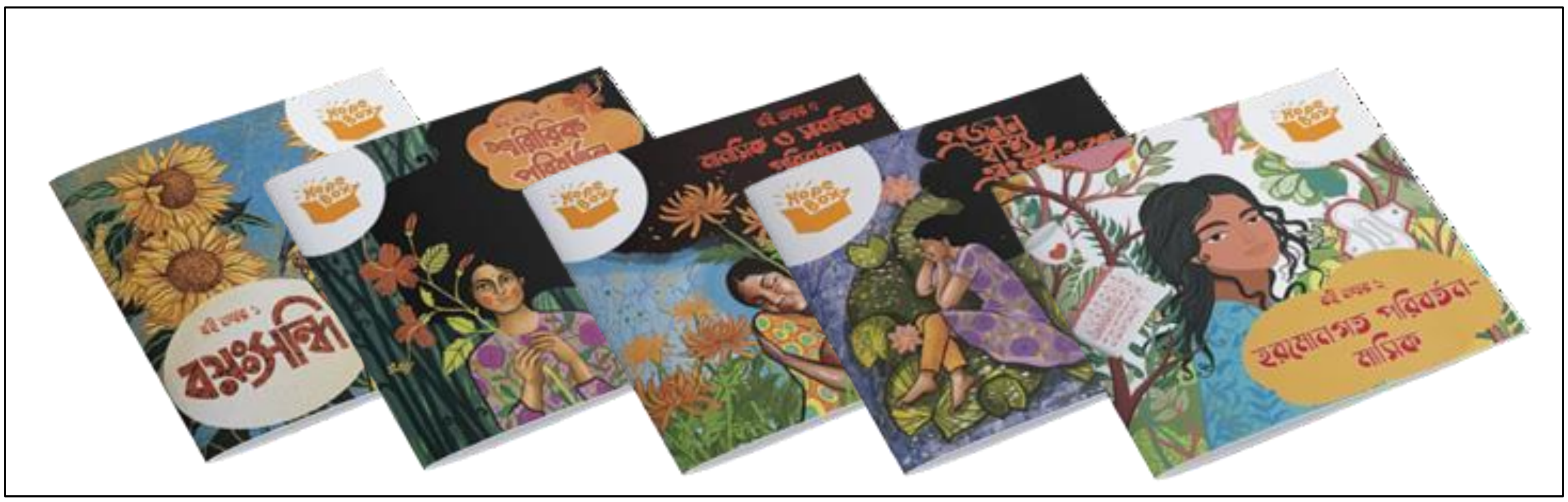

Postcards

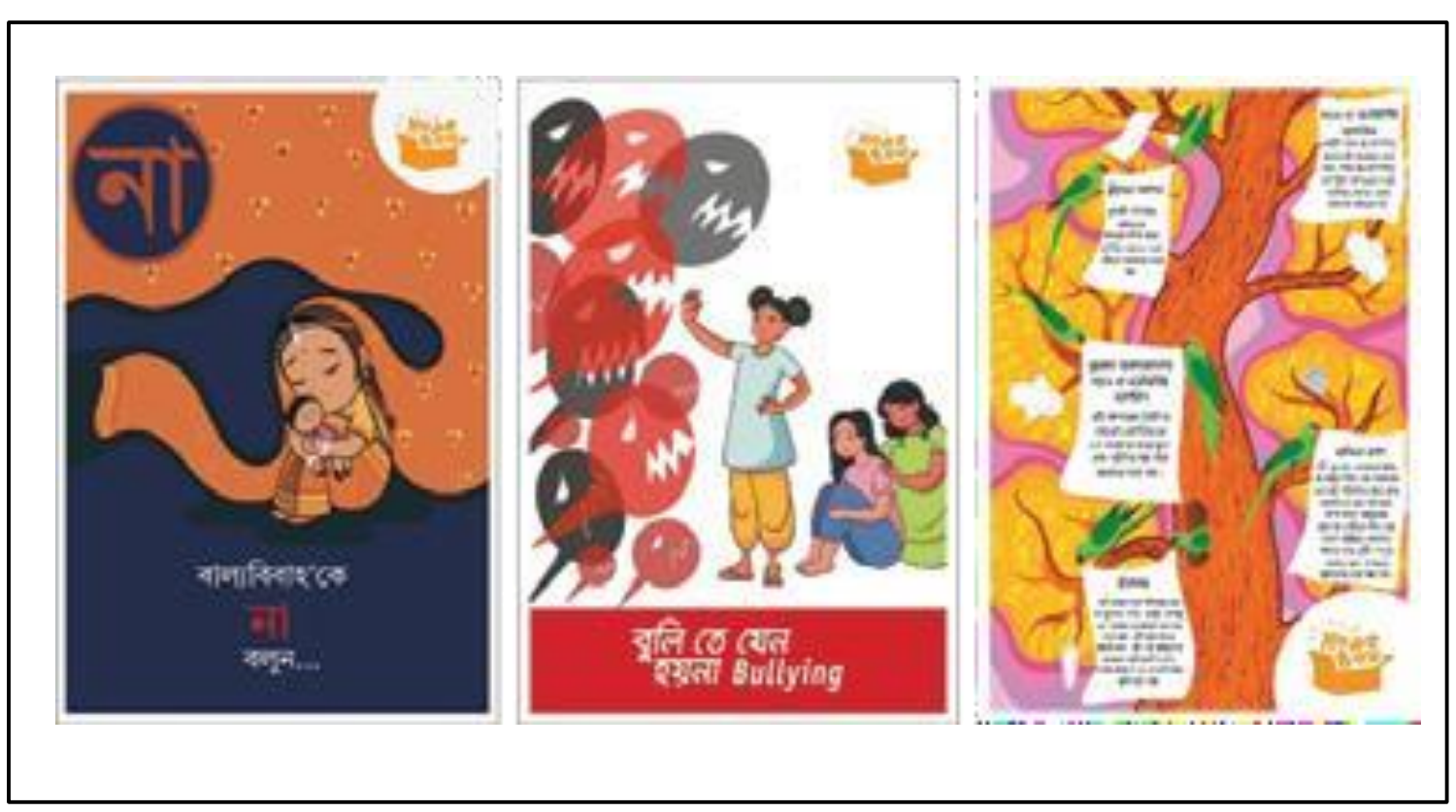

Puzzle

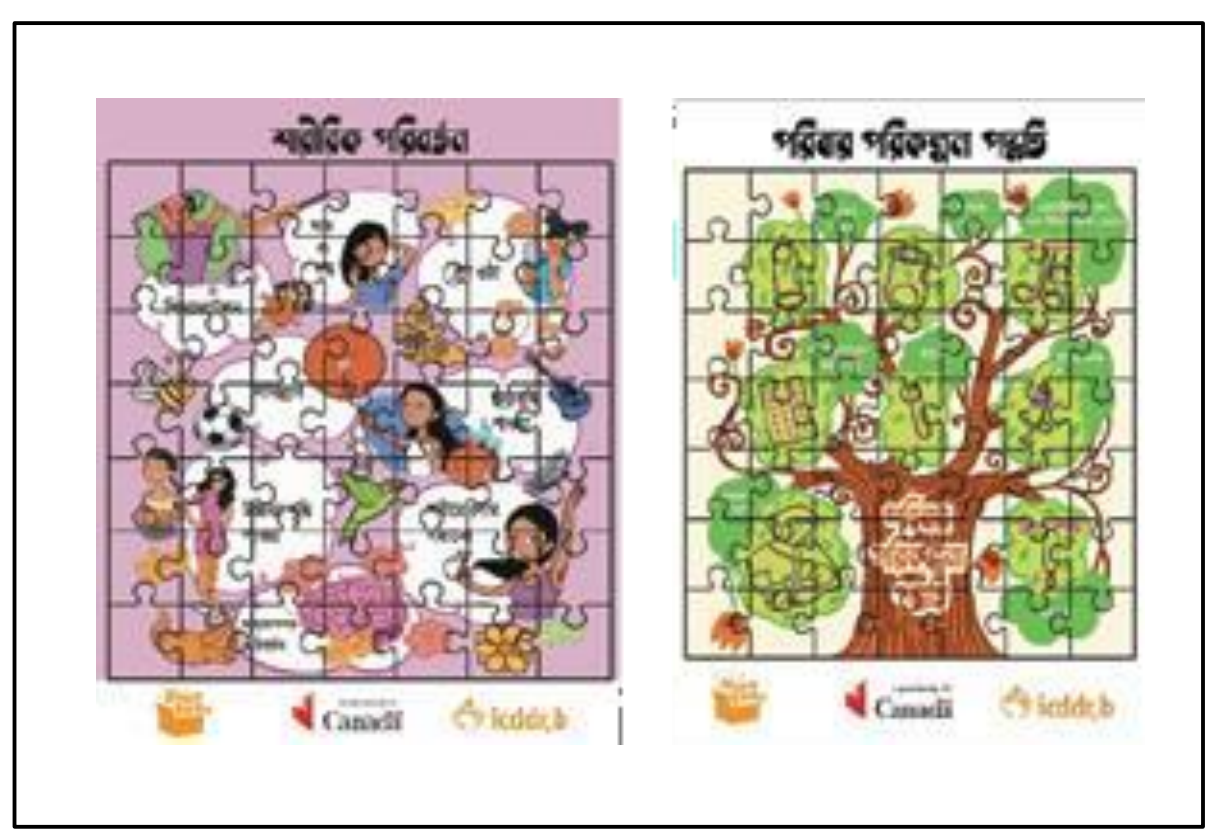

Figure S1. Components of “HopeBox

Table S1. Description of "HopeBox" components

No.	Component	Name	Description
1	Booklet	Booklet 01: Puberty	Overall, the content provided an understanding of the various changes and experiences that adolescents undergo during puberty.
		Booklet 02: Hormonal Changes – Menstruation	Explained hormonal changes during puberty, menstruation, menstrual cycle, and menstrual health and hygiene management.
		Booklet 03: Physical Changes	Covered general bodily changes which adolescent boys and girl go through during puberty.
		Booklet 04: Reproductive Health and Pregnancy	Provided information on reproductive health, pregnancy, and prevention methods.
		Booklet 05: Mental and Social Changes	Discussed mental health, and also emotional, psychological changes, during adolescence.
2	Puzzle	Puzzle 01: Contraceptive Methods	A tool that helped adolescents learn about different contraceptive methods in an interactive way by matching pieces with correct names and uses.
		Puzzle 02: Physical Changes	Designed to help adolescents recognize and name common physical changes during puberty by assembling and matching puzzle pieces.
3	Postcard	Postcard 01: Bullying	Encourages adolescents to stand up against bullying and speak out for themselves.
		Postcard 02: Child Marriage	Encourages adolescents to stand up against child marriage.
		Postcard 03: Menstrual Hygiene Solutions	Offered simple guidance on maintaining menstrual hygiene, choosing appropriate products, and managing periods safely.
4	Menstruation Tracking Calendar	Tracks menstrual cycles	Helped adolescents to record their menstrual cycle
